# Citrate‐assisted efficient local delivery of naked oligonucleotide into live mouse brain cells

**DOI:** 10.1111/cpr.12622

**Published:** 2019-05-07

**Authors:** Haibin Zhou, Shouhua Zhang, Fei Lv, Wenzhi Sun, Lihua Wang, Chunhai Fan, Jiang Li, Ji Hu

**Affiliations:** ^1^ Division of Physical Biology & Bioimaging Center Shanghai Synchrotron Radiation Facility, Shanghai Institute of Applied Physics Chinese Academy of Sciences Shanghai China; ^2^ University of Chinese Academy of Sciences Beijing China; ^3^ School of Life Science and Technology ShanghaiTech University Shanghai China; ^4^ iHuman Institute ShanghaiTech University Shanghai China; ^5^ Institute of Neuroscience Chinese Academy of Sciences Shanghai China; ^6^ Chinese Institute for Brain Research Beijing China

**Keywords:** neuronal transfection, ssDNA, stereotaxic injection, vector‐free

## Abstract

**Objectives:**

Synthetic oligonucleotides have shown promise in brain imaging. However, delivery of oligonucleotides into live brain cells remains challenging. In this study, we aim to develop a facile yet efficient strategy for local delivery of oligodeoxynucleotide (ODN) to neural cells in live adult mouse brain.

**Materials and methods:**

A fluorescence‐labelled ODN was diluted with sodium citrate buffer (100 mmol/L, pH = 3). One microlitre of the mixture was injected into a live adult mouse brain. Six hours later, we sacrificed the mouse and prepared brain slices for microscopic imaging.

**Results:**

We find that the use of sodium citrate buffer in the one‐shot local delivery can improve the diffusion and cell entry efficiency of the unmodified ODN for dozens of times. Only 1 pmol ODN leads to hundreds of positively transferred brain cells. We reason that this promotion is due to the local acidic condition created by the citrate buffer, which leads to the protonation of the ODN and some membrane proteins, thus reduces the Coulomb repulsion between the ODN and the cell membrane. Based on this strategy, we demonstrate fluorescent microscopic imaging of brain cells in different brain regions including striatum, cortex, hippocampus and midbrain.

**Conclusions:**

The citrate buffer can be used as an adjuvant for facile and effective local injection delivery of ODNs, which may provide a new tool for brain imaging.

## 1 INTRODUCTION

A variety of diseases in the central nervous system (CNS), such as brain glioma, Parkinson disease and Huntington's disease, is often characterized by altered cell proliferation and dysregulation of certain biomolecules.^1^, ^2^, ^3^, ^4^ Thus, imaging of brain cells with high spatial resolution is highly desirable.^5^, ^6^ In recent decades, synthesized single‐stranded short nucleic acids (oligonucleotides) have been intensively investigated as molecular probes^7^, ^8^, ^9^, ^10^, ^11^, ^12^, ^13^ or signal generator/amplifier^14^, ^15^, ^16^ for brain imaging, owing to their sequence‐dependent specificity in binding target molecules/cells or generating signals in situ.^17^ However, efficient delivery of synthesized oligonucleotides into live CNS remains a grand challenge, due to that the oligonucleotides (especially ssDNAs) can hardly be delivered by conventional viral vectors. Their poor structural stability, penetrability and cell uptake efficiency in the highly complex nervous system limit the use of direct local delivery.

One traditional approach for improving the oligonucleotide delivery efficiency is to use carriers like cationic lipids, polymers and nanoparticles, which help the anionic oligonucleotides overcome the Coulomb repulsion from the negatively charged cell membrane, thus favour the cell entry^10^, ^18^, ^19^, ^20^, ^21^; another approach is to covalently functionalize the oligonucleotides with chemical groups and molecules (eg, thiophosphorylation, 2′‐O‐methylation, 2′‐O‐methoxyethylation, 2′‐fluoro substitution, locked nucleic acid modification and peptide nucleic acid modification).^22^, ^23^ These strategies can strengthen the structural stability of the oligonucleotides and improve their efficiency to penetrate the physiological barriers and enter the target cells. However, these strategies still suffer from either low efficiency or inconvenience, which usually require high dose and repetitive administration of synthetic materials/chemicals.^27^, ^28^

Here in this study, we aim to develop a facile yet efficient strategy to deliver an oligodeoxynucleotide (ODN) into neural cells in live adult mouse brain. We find that proper local acidic condition created by citrate buffer can dramatically improve the entry efficiency of unmodified ODN into the brain cells. An injection of only 1 pmol of ODN can transfer hundreds of brain cells. Thus, the citrate buffer of appropriate concentration and pH can be used as an effective adjuvant for one‐shot local injection delivery of ODNs. Based on this strategy, we demonstrate microscopic imaging of brain cells in different brain regions using a fluorescence‐labelled ODN.

## 2 MATERIALS AND METHODS

### 2.1 Materials

Citrate and trisodium citrate dihydrate were obtained from Sinopharm Chemical Reagent Co., Ltd. The sodium citrate buffer (100 mmol/L, pH = 3) was prepared by mixing 100 mmol/L citrate acid and sodium citrate. The 28‐nt Cy3‐labelled ODN (5′‐3′ sequence: TTGCACATGCCGGAGCCGTTGTCGACGA) was synthesized and purified by Sangon Biotech (China). All chemicals and reagents were of analytical grade.

### 2.2 Animals

Adult male C57BL/6 mice (purchased from Shanghai Biomodel Organism Science & Technology Development Co., Ltd.) were used in this study. The animals were housed in cages under standard laboratory conditions (12:12 hour light‐dark cycle, 20 ± 1°C, food and water ad libitum). All experimental procedures were performed according to institutional guidelines.

### 2.3 Stereotaxic injection

The ODN (1 μmol/L) dissolved in sodium citrate buffer (typically 100 mmol/L, pH = 3) before injection. Micropipettes for an auto‐injector Nanoject III (Drummond), with a tip diameter of 20‐50 μm, were made on a microelectrode puller (Narishige PP‐830). Micropipettes filled with Ringer's solution were attached to the auto‐injector, and a few microlitres of samples were sucked in from the tip. For injection, adult mice fixed to a stereotaxic apparatus were anaesthetized with 1% isoflurane via continuous delivery through a nose cone. With the animal lying on a heating pad, 1 μL of samples was injected over 15 minute. The head wound was sutured at the end of the experiment, and mice were kept on a heating pad until full recovery. The injection sites were stereotactically placed to midbrain (AP: −3.2, ML: ±1.0, DV: −3.6) and cortex (AP: +0.4, ML: ±1.6, DV: −1.8), striatum (AP: 0, ML: ±2.0, DV: −3.0) and hippocampus (AP: −2.0, ML: ±1.2, DV: −2.0) relative to bregma.

### 2.4 Perfusion and tissue preparation

Six hours after injection, the animals were anaesthetized with an overdose of 1% sodium pentobarbital solution and intracardially perfused with 4% paraformaldehyde in phosphate‐buffered saline (PBS, pH = 7.4). Brains were dissected and post‐fixed in the same fixative for 2 hours and immersed in 30% sucrose solution at room temperature overnight. Mouse brains were cut on a freezing microtome (LEICA CM3050 S) in the coronal plane (50 µm thickness). All sections were collected on microslides, washed three times for 5 minute each in PBS and mounted with a mounting agent with DAPI (4′,6‐diamidino‐2‐phenylindole, dihydrochloride, 1 µg/mL) and 50% (v/v) glycerol in PBS.

### 2.5 Imaging and data analysis

Images of brain sections were acquired using a Nikon AR1 confocal microscope. Representative sections from three cases of each condition covering the centre of injection sites were imaged. Delivery area and cell counts were estimated using ImageJ software. Briefly, the area with fluorescence above the threshold (mean background) was calculated, and all cells within the delivery area were counted with ImageJ. Data were analysed using one‐way ANOVA followed by Turkey's *post hoc* test. *P* < 0.05 was considered statistically significant.

## 3 RESULTS

**Figure 1:**
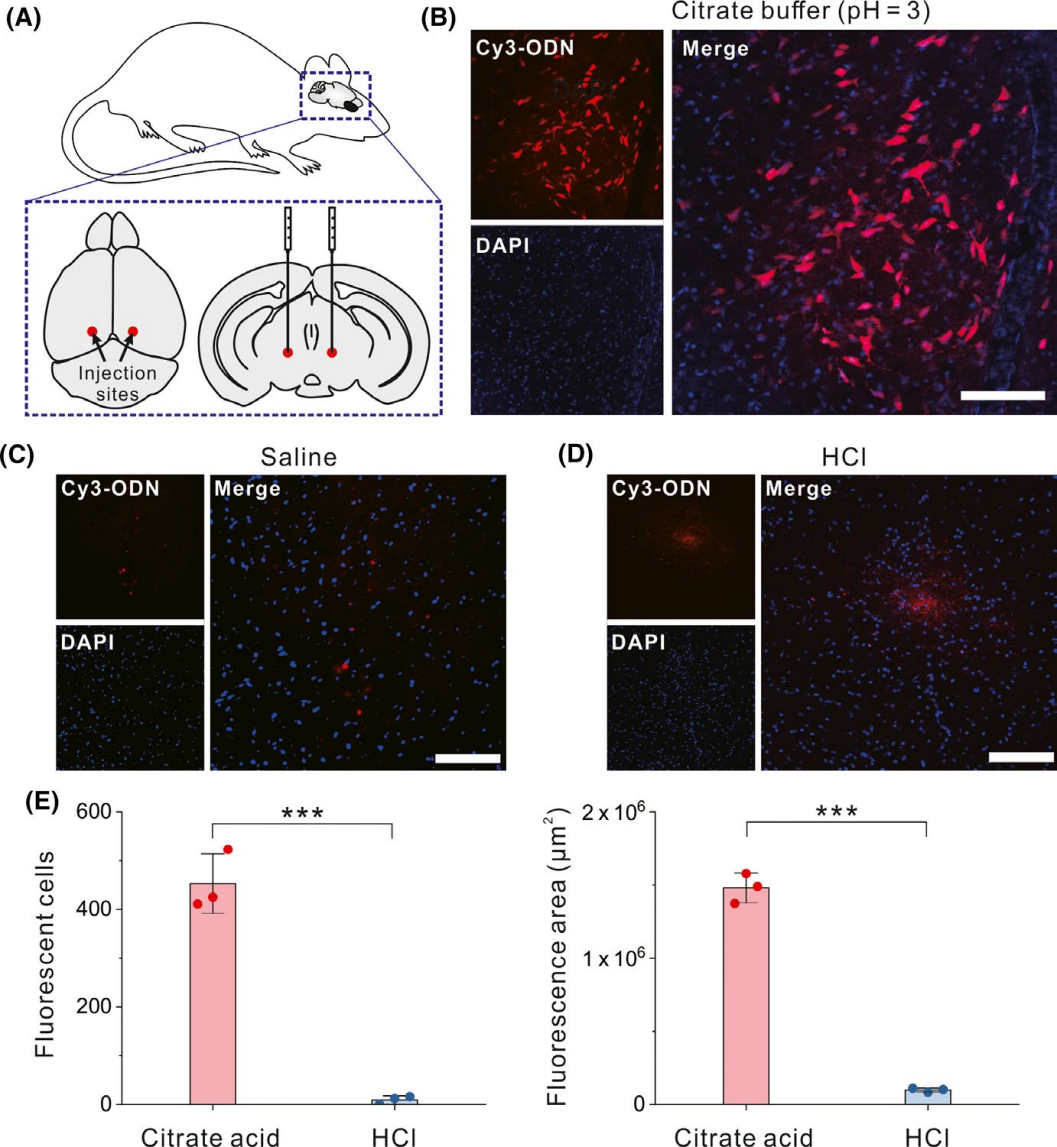
Citrate acid‐assisted local delivery of ODNs into live mouse brain cells. A, Schematic of the injection in the mouse brain. B‐D, Confocal microscopic images of the mouse brain slices after the injection of Cy3‐labelled ODNs assisted with citrate acid, normal saline or HCl, respectively. Scale bar, 100 μm. E, Counts of positively transfected cells (left) and measured diffusion area of the fluorescence (right). Error bars represent the standard deviation from three independent injections. ***P < 0.01

We first test whether the citrate acid can promote the cell entry of ODNs in the mouse brain. We mixed a Cy3‐labelled 28‐nt ODN with citrate acid solution (100 mmol/L, pH = 3) and carried out a stereotactic injection of the mixture (1 µL each injection) into the brain parenchyma of live mice (Schematic shown in Figure 1A). Six hours after the injection, we sacrificed the mice and prepared 50 µm thick brain slices for confocal microscopic imaging. As shown in Figure 1B, around the injection spot in the midbrain, more than 400 cells on average showed bright fluorescence (Figure 1B,E), indicating that a considerable number of cells are positively transfected with the fluorescent ODNs. In contrast, using normal saline alone to assist ODN injection did not result in obvious fluorescence in cells (<10 positive cells, shown in Figure 1C). Meanwhile, using hydrochloric acid (HCl, also pH = 3) instead of citrate acid also led to few fluorescent cells (<10 on average), and the fluorescent ODNs were mostly distributed in the extracellular space (ECS; Figure 1D,E). We also found that the citrate‐assisted injection led to a ~15‐fold larger fluorescence diffusion area compared with which used HCl (Figure 1E), which also reflects the improved delivery efficiency resulted from the citrate. Based on the above results, we reason that an acidic local environment around the injection site favours the diffusion and cell entry of the ODNs. Although the HCl solution is also acidic, it lacks the buffering capacity to maintain the acidity of the local environment. In contrary, citrate is an excellent pH buffer, which might contribute to the difference in delivery efficiency between using citrate and using HCl.

**Figure 2:**
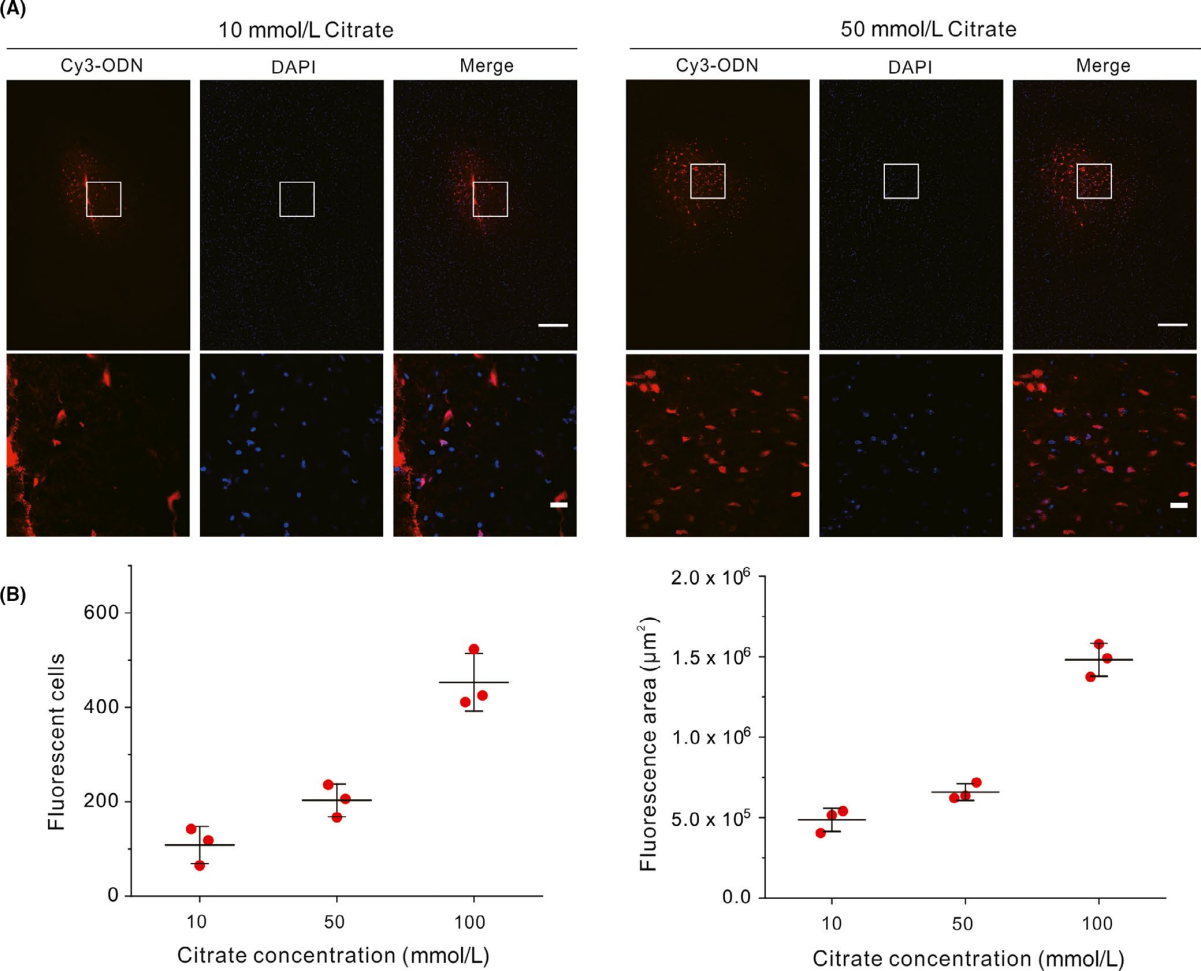
Dependency of delivery efficiency on citrate concentration. A, Confocal images of brain samples treated with citrate buffers of different concentrations (left, 10 mmol/L; right, 50 mmol/L). Upper: full view around the injection sites (scale bar, 200 μm). Lower: magnification (scale bar, 20 μm) of the marked regions from the upper. B, Counts of positively transfected cells (left) and measured diffusion area of the fluorescence (right). Error bars represent the standard deviation from three independent injections

Next, we studied the effect of buffering capacity on delivery efficiency. Given that the buffering capacity is largely determined by the buffer concentration, we prepared citrate buffer solutions with identical pH (pH = 3) but different citrate concentrations (10, 50 mmol/L) for comparison. As results, we found that the 10 mmol/L citrate buffer led to few fluorescent cells and low fluorescence diffusion around the injection site, while the 100 mmol/L citrate buffer resulted in the highest diffusion and cell entry efficiency, indicating that the delivery efficiency increases along with the increase of buffer concentration within the range we tested (Figure 2). These results further verify that an adequate buffering capacity to maintain the acidic local environment is necessary for efficient delivery of ODNs.

**Figure 3:**
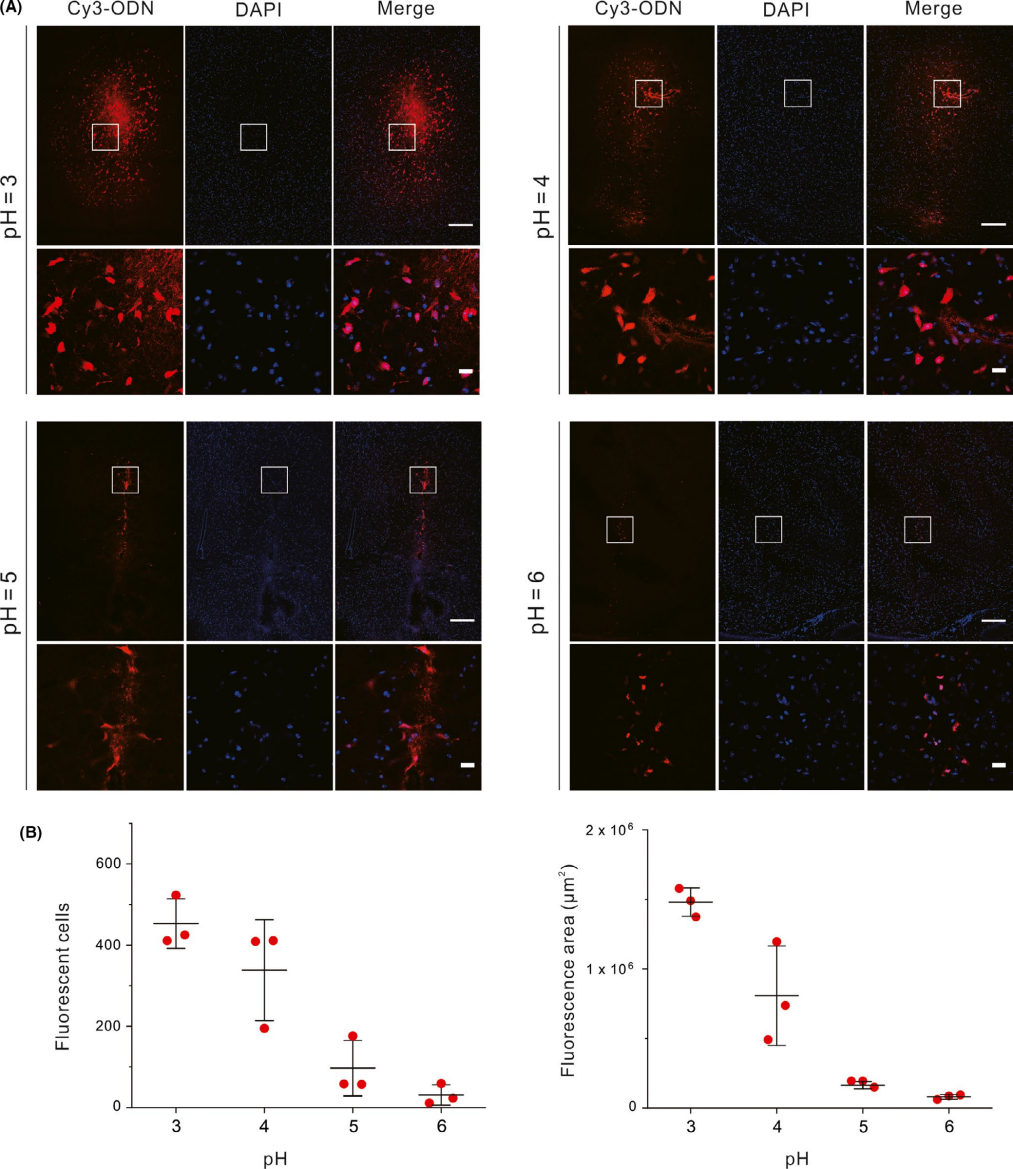
Dependency of delivery efficiency on the pH of the citrate buffer. A, Confocal microscopic images of brain samples treated with citrate buffers of different pH (pH = 3, 4, 5 or 6, respectively). Upper: full view around the injection sites (scale bar, 200 μm). Lower: magnification (scale bar, 20 μm) of the marked regions from the upper. B, Counts of positively transfected cells (left) and measured diffusion area of the fluorescence (right). Error bars represent the standard deviation from three independent injections

We further investigated the effect of pH value on the brain cell delivery of the ODN. We prepared a series of citrate buffer solutions with identical citrate concentration (100 mmol/L) but different pH (pH = 3, 4, 5 or 6, adjusted with NaOH, respectively), which were then used to assist the ODN injection. As results, we found that the numbers of fluorescent cells, as well as the fluorescence area, decreased along with the increase of pH (Figure 3). When the pH value was up to 6, few positively transfected cells can be observed. Within the range we tested, pH = 3 was the optimal pH value for the delivery of ODNs. It is known that DNA molecule and cell membrane are both negatively charged under the neutral condition, leading to Coulomb repulsion that hampers the cell entry of DNA. We reason that the acidic environment can protonate the DNA bases (with an estimated isoelectric point of about 4~5^29^) and some membrane proteins (with isoelectric points <7),^30^ thus reduces the overall Coulomb repulsion.^31^ Moreover, compared with buffers of higher pH, the buffer of pH 3 can also provide a higher buffer capacity to maintain the acidic environment. These factors may together contribute to the improved ODN delivery. However, according to published literature, reducing the pH further is unlikely to be feasible considering DNA depurination and cleavage reactions at extremely low pH.^32^, ^33^

**Figure 4:**
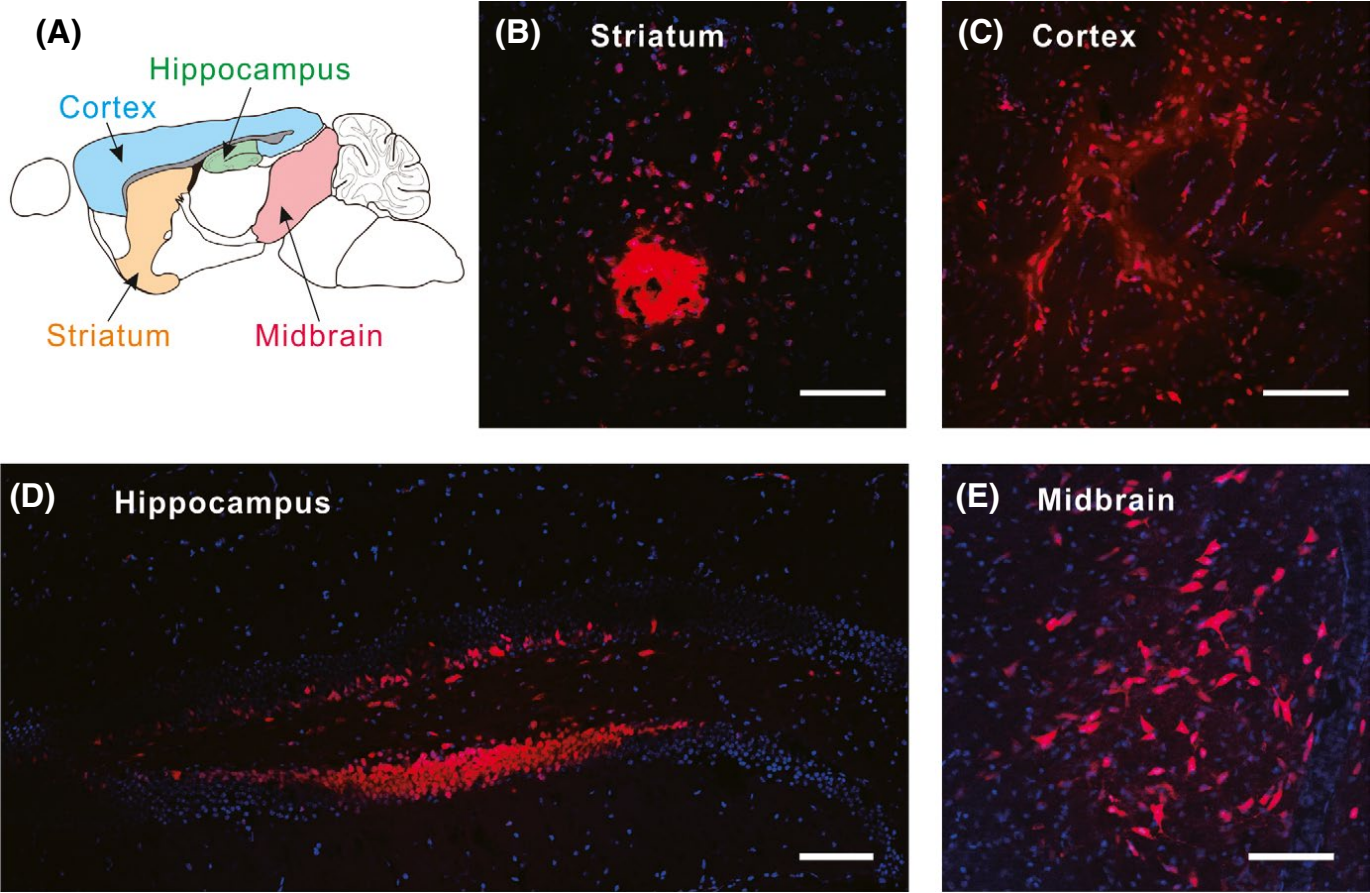
Delivery of ODNs to different brain regions. A, Cartoon of the target regions in the mouse brain. B‐E, Confocal microscopic images of slides from striatum, cortex, hippocampus and midbrain, respectively. Scale bars, 100 μm

The brain is highly heterogeneous in structure and function (Figure 4A). To demonstrate the generality of this delivery strategy, we performed the injections towards different brain regions. Here, the Cy3‐labelled ODN mixed with citrate buffer (100 mmol/L, pH = 3) was injected into four different brain regions, including striatum, cortex, hippocampus and midbrain, respectively. As results (Figure 4B‐E), all the samples presented plenty of fluorescent cells around the injection sites. As the control, injections of the ODN at these regions with normal saline did not result in widespread fluorescent cells (Figure S1). These results indicate that citrate‐assisted delivery strategy can be applied in different brain regions. Notably, the morphologies of the fluorescent cells in these regions seemed normal, suggesting minimal cell damage resulted from this local delivery strategy. Thus, this strategy holds potential in the delivery of ODN probes for nucleic acid imaging in brain cells.

## 4 DISCUSSION

In this study, we have developed a facile while efficient local injection strategy to deliver naked ODNs into live mouse brain cells. This strategy is based on the use of a citrate buffer solution with pH 3 as an adjuvant, which can improve the diffusion and cell entry efficiency of ODNs for dozens of times in the mouse brain. An injection of 1 pmol ODN resulted in hundreds of positively transferred brain cells. This effect might be attributed to the acidic local environment created by the citrate buffer, which has a high buffering capacity. The acidic environment may protonate the DNA bases and some membrane proteins, thus favours the cellular uptake of ODNs. Although the acidic condition has been used to assist assembly of ODNs with anionic nanomaterials,^32^, ^33^ its use in assisting cellular delivery of ODNs has not been documented, to our knowledge. We demonstrate that this simple method can be applied in the delivery of ODNs towards different brain regions, including striatum, cortex, hippocampus and midbrain, with negligible cell damage. In future studies, the effects of citrate buffer on the viability and functionality of brain cells are to be systematically investigated. We envision that this strategy can be used in delivering ODNs for brain imaging or molecular diagnosis in live brain cells.

## CONFLICT OF INTEREST

The authors declare no conflicts of interests.

## AUTHOR CONTRIBUTION

WS, LW, CF, JL and JH designed the experiments, supervised the study and revised the paper; HZ, SZ and FL conducted the experiments, analysed the data and wrote the draft of the manuscript. All authors have reviewed the final version of the manuscript and approve it for publication.
