# Supplementary material for: Citrate‐assisted efficient local delivery of naked oligonucleotide into live mouse brain cells
Source: Cell Prolif. 2019 May 7;52(4):e12622. doi: 10.1111/cpr.12622 (PMC6668962; doi:10.1111/cpr.12622)
Supplement: Supplementary file 1 [file CPR-52-e12622-s001.docx]

**Supporting information**

**Citrate-assisted efficient local delivery of naked oligonucleotide into live mouse brain cells**

**Haibin Zhou^1,2,3^ | Shouhua Zhang^1,2,3^ | Fei Lv^2,3,4,5^ | Wenzhi Sun^3,4,6^ | Lihua Wang^1^ | Chunhai Fan^1^ | Jiang Li^1*^ | Ji Hu^3*^**

^1^ Division of Physical Biology & Bioimaging Center, Shanghai Synchrotron Radiation Facility, Shanghai Institute of Applied Physics, Chinese Academy of Sciences, Shanghai 201800, China

^2^ University of Chinese Academy of Sciences, Beijing 100049, China

^3^ School of Life Science and Technology, ShanghaiTech University, Shanghai 201210, China

^4^ iHuman Institute, ShanghaiTech University, Shanghai 201210, China;

^5^ Institute of Neuroscience, Chinese Academy of Sciences, Shanghai 200031, China;

^6^ Chinese Institute for Brain Research, Beijing 102206, China.

***Correspondence**

Jiang Li, Division of Physical Biology & Bioimaging Center, Shanghai Synchrotron Radiation Facility, Shanghai Institute of Applied Physics, Chinese Academy of Sciences, Shanghai 201800, China.

E-mail: lijiang@sinap.ac.cn

and

Ji Hu, School of Life Science and Technology, ShanghaiTech University, Shanghai 201210, China.

Email: huji@shanghaitech.edu.cn

**SUPPLEMENTAL FIGURES**


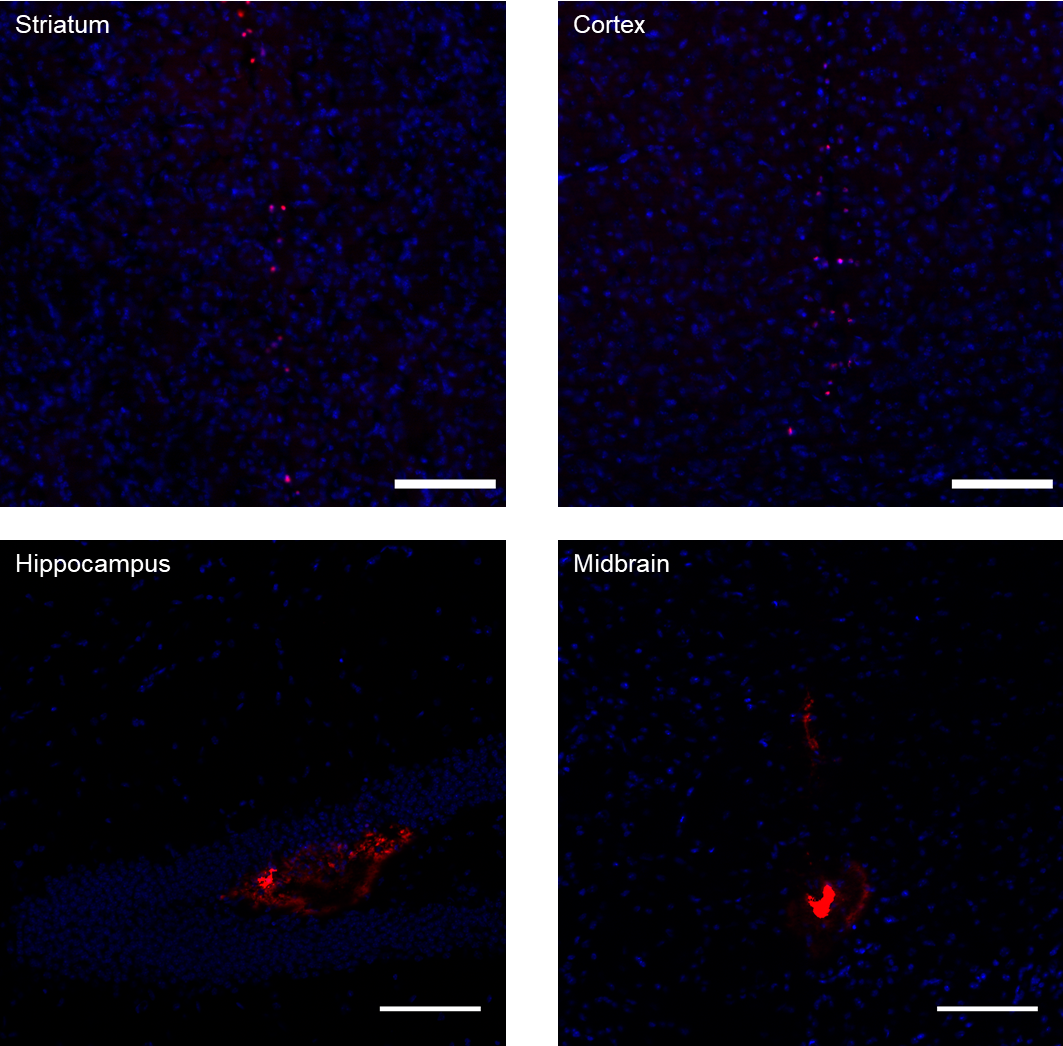


**Figure S1.** Fluorescence images of control samples (striatum, cortex, hippocampus, and midbrain, respectively) undergone ODN local delivery with normal saline. Scale bars, 100 µm.
